# EyeG2P: an automated variant filtering approach improves efficiency of diagnostic genomic testing for inherited ophthalmic disorders

**DOI:** 10.1136/jmg-2022-108618

**Published:** 2023-01-20

**Authors:** Eva Lenassi, Ana Carvalho, Anja Thormann, Liam Abrahams, Gavin Arno, Tracy Fletcher, Claire Hardcastle, Javier Lopez, Sarah E Hunt, Patrick Short, Panagiotis I Sergouniotis, Michel Michaelides, Andrew Webster, Fiona Cunningham, Simon C Ramsden, Dalia Kasperaviciute, David R Fitzpatrick, Genomics England Research Consortium, Graeme C Black, Jamie M Ellingford

**Affiliations:** 1 Division of Evolution, Infection and Genomic Sciences, School of Biological Sciences, Faculty of Biology, Medicine and Health, University of Manchester, Manchester, UK; 2 Manchester Centre for Genomic Medicine, St Mary's Hospital, Manchester University NHS Foundation Trust, Manchester, UK; 3 Manchester Royal Eye Hospital, Manchester University NHS Foundation Trust, Manchester, UK; 4 MRC Human Genetics Unit, MRC Institute of Genetics and Cancer, University of Edinburgh, Edinburgh, UK; 5 Medical Genetic Unit, Pediatric Hospital, Coimbra Hospital and Universitary Centre (CHUC), Coimbra, Portugal; 6 European Molecular Biology Laboratory, European Bioinformatics Institute, Cambridge, UK; 7 Genomics England Ltd, London, UK; 8 UCL Institute of Ophthalmology, University College London, London, UK; 9 Department of Ophthalmology, Moorfields Eye Hospital NHS Foundation Trust, London, UK; 10 Sano Genetics Ltd, Cambridge, UK

**Keywords:** Genomics, Eye Diseases, Genetic Variation

## Abstract

**Background:**

Genomic variant prioritisation is one of the most significant bottlenecks to mainstream genomic testing in healthcare. Tools to improve precision while ensuring high recall are critical to successful mainstream clinical genomic testing, in particular for whole genome sequencing where millions of variants must be considered for each patient.

**Methods:**

We developed EyeG2P, a publicly available database and web application using the Ensembl Variant Effect Predictor. EyeG2P is tailored for efficient variant prioritisation for individuals with inherited ophthalmic conditions. We assessed the sensitivity of EyeG2P in 1234 individuals with a broad range of eye conditions who had previously received a confirmed molecular diagnosis through routine genomic diagnostic approaches. For a prospective cohort of 83 individuals, we assessed the precision of EyeG2P in comparison with routine diagnostic approaches. For 10 additional individuals, we assessed the utility of EyeG2P for whole genome analysis.

**Results:**

EyeG2P had 99.5% sensitivity for genomic variants previously identified as clinically relevant through routine diagnostic analysis (n=1234 individuals). Prospectively, EyeG2P enabled a significant increase in precision (35% on average) in comparison with routine testing strategies (p<0.001). We demonstrate that incorporation of EyeG2P into whole genome sequencing analysis strategies can reduce the number of variants for analysis to six variants, on average, while maintaining high diagnostic yield.

**Conclusion:**

Automated filtering of genomic variants through EyeG2P can increase the efficiency of diagnostic testing for individuals with a broad range of inherited ophthalmic disorders.

**WHAT IS ALREADY KNOWN ON THIS TOPIC**

- Genome diagnostics for ophthalmic disorders have clinical utility, but automated prioritisation of genomic variants that underpin ophthalmic disorders remains a challenge in clinical diagnostics.
- This is further emphasised by the spectrum and diversity of disease-causing genomic variants that have been described to underpin these heterogeneous conditions.

**WHAT THIS STUDY ADDS**

- We compare EyeG2P, an updated software tool available through the Ensembl Variant Effect Predictor, with other routine approaches for variant prioritisation in the diagnosis of genomic ophthalmic disorders.
- We show that EyeG2P can achieve high sensitivity to disease-causing variations with improvements in the precision of variant analysis to routine approaches.

**HOW THIS STUDY MIGHT AFFECT RESEARCH, PRACTICE OR POLICY**

- Our data support the use of EyeG2P as a front-line software tool for analysis of genomic data sets for individuals with suspected genomic ophthalmic disorders.

## Introduction

Inherited ophthalmic disorders are a clinically and genetically heterogeneous group of conditions that represent a major cause of blindness in children and working-age adults.^1–3^ They include developmental disorders affecting the whole eye (eg, microphthalmia and anophthalmia, aniridia), childhood cataracts, inherited retinal disorders (eg, rod-cone dystrophies, cone-rod dystrophies, macular dystrophies, Leber congenital amaurosis) and inherited optic neuropathies (eg, Leber hereditary optic neuropathy, dominant optic atrophy). Notably, these conditions may present in isolation or as part of a syndrome with major extraocular features (eg, Usher syndrome, Joubert syndrome, Bardet-Biedl syndrome).

Obtaining a genetic diagnosis in individuals with inherited ophthalmic disorders has been shown to inform management and to facilitate genetic counselling.^3^ As a result, genomic investigations are increasingly being used as a front-line diagnostic tool for this group of conditions.^4–9^ The more widespread availability of preimplantation genetic testing and gene-directed interventions (eg, gene therapy for Leber congenital amaurosis due to pathogenic genetic variation impacting *RPE65*) has increased both the value and the risk of genetic testing, placing ever increasing demands on the delivery of genetic testing in a timely and accurate manner.^10–15^

In this study, we evaluate the diagnostic utility of EyeG2P, a publicly accessible tool for prioritised analysis of variants identified in genes known to cause inherited ophthalmic conditions. We curated disease-related genes through robust and transparent standards and assessed the sensitivity and precision of EyeG2P both retrospectively and prospectively. EyeG2P uses logical filtering of genomic variants, taking into account their predicted molecular consequence, their population frequency and prior knowledge of disease mechanisms and inheritance patterns. Overall, we show that using EyeG2P as a first-tier analytical strategy reduces the number of variants requiring analysis by clinical scientists and hence increases the precision and efficiency of diagnostic testing. We demonstrate the utility of EyeG2P both for targeted testing and whole genome-based sequencing approaches, and compare diagnostic yields and precision with those achieved by Exomiser and Genomics England tiering approaches for individuals with genomic data sets.

## Methods

### Curation of known disease genes

The gene2phenotype (G2P) web portal (https://www.ebi.ac.uk/gene2phenotype/)^16^ was used to develop and curate an ophthalmic disorders panel. For the purpose of this work, we defined inherited ophthalmic disorders as a group of predominantly monogenic conditions that affect the eye. This group encompasses diseases affecting the anterior segment, cornea, lens, vitreous, retina and optic nerve, but not disorders in which the main site of dysfunction is, for example, the eyelid or the visual cortex. New entries were initiated by selection of a relevant gene symbol from the list of preloaded genes (with their associated Ensembl identifiers). For each entry, a gene or locus was linked via a disease mechanism to a disease. These connections were made after inspecting MEDLINE (through the PubMed interface); search terms included the gene name (HUGO Gene Nomenclature Committee, HGNC) and the disease name (as a minimum). A disease mechanism was defined as both an allelic requirement (mode of inheritance; eg, biallelic or monoallelic) and a variant consequence (mode of pathogenicity; eg, loss-of-function). A confidence attribute (confirmed, probable or possible) was also assigned to indicate how likely it is that the gene is implicated in the cause of the disease; the rules used to assign confidence, allelic requirement and variant consequence to entries have been previously described.^16^ Each *locus–genotype–mechanism–disease–evidence* link was further characterised by assigning to it a set of phenotype terms (ie, clinical signs and symptoms) from the Human Phenotype Ontology.^17^ The identifiers of the relevant publications that provide evidence for a specific gene–disease thread were stored and are available through the G2P web portal.

### Sequencing and variant identification

Genomic sequencing data sets were generated in a tertiary healthcare setting (North West Genomic Laboratory Hub, Manchester, UK; ISO 15189:2012, United Kingdom Accrediation Service medical reference 9865) or through the Genomics England 100,000 Genomes Project. Individuals provided written consent for genomic analysis. All clinical data were collected as part of routine clinical care.

#### Diagnostic gene panels

Routine diagnostic gene panel testing was performed as previously described.^4 6 18^ Briefly, enrichment techniques were used to select specific genes for analysis using Agilent SureSelect (Agilent Technologies, Santa, Clara, California) target enrichment kits that were designed to capture selected intronic regions and all protein-coding exons±50 bp of flanking intronic sequences of the selected panels of known disease genes. The decision on which panel to use was made by the referring clinician (either a consultant ophthalmologist or a consultant clinical geneticist with an interest in ophthalmic genetics).

High-throughput genomic sequencing was performed using Illumina HiSeq and NextSeq platforms. Raw sequencing reads were first aligned to the GRCh37 reference genome using Burrow-Wheeler Aligner (BWA)-mem,^19^ with single nucleotide variants (SNVs) and small insertions/deletions (indels) identified using the Genome Analysis Toolkit (GATK).^20^ Larger and more complex indels were identified using Pindel, and CNVs were identified using DeCON.^21^ Variants were filtered using quality and read depth thresholds, as well as inhouse allele frequencies. The zygosity of CNVs was estimated based on their relative read depths. Regions that are highly polymorphic and/or difficult to survey through short-read, high-throughput techniques were masked from the initial analysis, specifically *RP1L1* exon 4, *USH1C* exon 18 and *RPGR* orf15.

#### Whole genome sequencing

Whole genome sequencing data sets were generated as part of the Genomics England 100,000 Genomes Project.^22^ Briefly, this involved complete sequencing of the accessible genome at a target coverage of 30× per nucleotide using Illumina sequencing technology. Alignment of sequencing reads and variant calling was performed through an Illumina pipeline involving Isaac aligner, Starling (for SNVs and indels), Canvas and Manta (for structural variants and CNVs). Further details of these pipelines are available at https://research-help.genomicsengland.co.uk/display/GERE/Genomic+data.

### Variant analysis

#### Routine diagnostics for gene panel analysis

Routine genomic analysis was performed using the Congenica platform.^23^ This process involves filtering variants based on gene/location depending on the gene panel applied, the population frequency and the predicted molecular consequence. A complete list of the criteria used for variant filtering is available in online supplemental tables 1 and 2. After prefiltering, variants were analysed by clinically accredited scientists and were classified in accordance with the 2015 American College of Medical Genetics and Genomics and Association for Molecular Pathology (ACMG/AMP) best practice guidelines.^24 25^

#### EyeG2P analysis

We merged variant calls for SNVs, indels and CNVs into single Variant Call Format (VCF) files for each individual and annotated them using the G2P plugin for Ensembl Variant Effect Predictor.^16 26^ This plugin requires an input file which lists genes of interest and their allelic requirements; we used the EyeG2P data set and an allele frequency cut-off of 0.001 for variants in monoallelic genes and 0.05 for variants in biallelic genes. An additional list was used as input including all ClinVar pathogenic or likely pathogenic variants, all variants predicted to have a significant impact on splicing by SpliceAI,^27^ and a selection of hypomorphic alleles that are known to be pathogenic but exceed the variant frequency thresholds specified.

#### Comparisons between routine diagnostic analysis and EyeG2P

Results from EyeG2P were retrospectively compared with clinically reported variants identified from routine diagnostic analysis in 1234 individuals with inherited ophthalmic conditions. All the relevant study participants had a confirmed (or a provisional) molecular diagnosis and carried pathogenic, likely pathogenic or variants of uncertain significance, in accordance with the guidelines proposed by the ACMG/AMP^21^; these changes were identified in a disease-causing state and were deemed to fully account for the patient’s phenotype at the time of routine diagnostic analysis. Prospectively collected data from 83 individuals were also used for comparison. The sensitivity and precision of EyeG2P in comparison with results from routine diagnostic analysis were subsequently assessed and the 95% CI calculated through Bayesian inference using the *binom* R package. All statistical analyses were performed in R and graphics created in R and BioRender.

#### EyeG2P applied to whole genome data sets

Ten individuals recruited to the Genomics England 100,000 Genomes Project^22^ with ophthalmic disorders were selected for analysis using EyeG2P (six cases with rod-cone dystrophies, one with a rod dysfunction disorder and three with macular dystrophies).

Genomic variants identified for each of these 10 individuals were accessed (Genomics England Research Registry ID: RR117) and variants were prioritised in four distinct phases: (1) all SNVs, indels and CNVs/structural variants impacting genes within the EyeG2P gene panel were selected; (2) variants identified as high quality, defined as those variants also present in an aggregated file of high-quality variants for the 100,000 Genomes Project cohort; (3) variants matching the user-specified criteria for EyeG2P (as described in the EyeG2P analysis section); and (4) subsequent filtering to keep only variants impacting the most important (canonical) transcripts for genes and present in fewer than 4 of the 10 individuals analysed. Variants prioritised by this process were compared with clinically reported variants available within the gmc_outcome_questionnaire in the Genomics England Research Environment from first-tier analysis of the genomic data performed by clinical scientists in UK Genome Medicine centres. Data were compared with variants prioritised by Exomiser and Genomics England tiering variant analysis approaches that were applied during the 100,000 Genomes Project.^22^ We also applied an updated version of tiering to these 10 cases, which is now available through the NHS Genomic Medicine Service, and includes consideration of variants in ClinVar and triaging of CNVs (TierA and TierNull). Briefly, SNVs and indels are tiered into three discrete groups (tier 1, tier 2 and tier 3). Variants in all three groups are rare in the population (max allele frequencies are set for dominant, recessive and mitochondrial inherited disorders and variants compared with frequencies available for internal cohorts, and cohort subsets in UK10K, Genome Aggregation Database and 1kG_phase3), have a protein-altering consequence in at least one transcript (high or moderate impact) and segregate appropriately within the family (this strategy can be relaxed to incomplete penetrance). Tier 1 and 2 variants impact genes within the virtual gene panel that is applied during analysis of the genomic data, in this case the retinal disorders gene panel available through PanelApp (https://panelapp.genomicsengland.co.uk/panels/307/): Tier 1, high-impact variants (predicted loss-of-function), de novo variants in monoallelic disorders and variants in ClinVar (or have the same amino acid change as a variant in ClinVar) with at least one pathogenic or likely pathogenic assertion; Tier 2, moderate-impact variants (eg, missense variants and splice region variants); and Tier 3, includes high-impact or moderate-impact variants that are outside the virtual gene panel applied during analysis. CNVs are tiered separately into two distinct groups: TierA, high-quality CNVs that overlap with genes or regions defined in the applied virtual gene panel; TierNull, high-quality CNVs (>2 kb) that do not overlap with the applied virtual gene panel.

## Results

### Curation of the literature identified 667 genes for inclusion in EyeG2P

Between April 2017 and June 2020, we interrogated the biomedical literature for genes associated with highly penetrant inherited ophthalmic disorders. We identified 667 unique disease-implicated genes, encompassing 564 MIM disease terms. Using evidence of gene–disease associations from 1624 scientific publications, we identified 559 as ‘confirmed’, 135 as ‘probable’ and 108 as ‘possible’. Within the 559 confirmed gene–disease pairs, the associated inheritance patterns were autosomal dominant (n=155), autosomal recessive (n=341), X linked (n=31), and other patterns including both autosomal dominant and recessive (n=32). A high-level assessment of the biological disease mechanism was performed for each gene–disease combination; 405 disorders were catagorised as loss-of-function disorders, 19 as dominant negative disorders and 62 as disorders exclusively associated with specific missense or inframe indel variants.

For each gene–disease pairs, the predominant compartment of the eye was determined: retina (n=303), lens (n=120), cornea (n=65), vitreous (n=22) and optic nerve (n=39). Notably, 245 of the curated pairs (37%) were associated with multisystemic disorders. Skeletal (n=97), skin (n=49), ear (n=63), kidney (n=48) and metabolism (n=39) were the most frequently associated extraocular manifestations.

### EyeG2P is highly sensitive for detection of pathogenic variants

**Figure 1:**
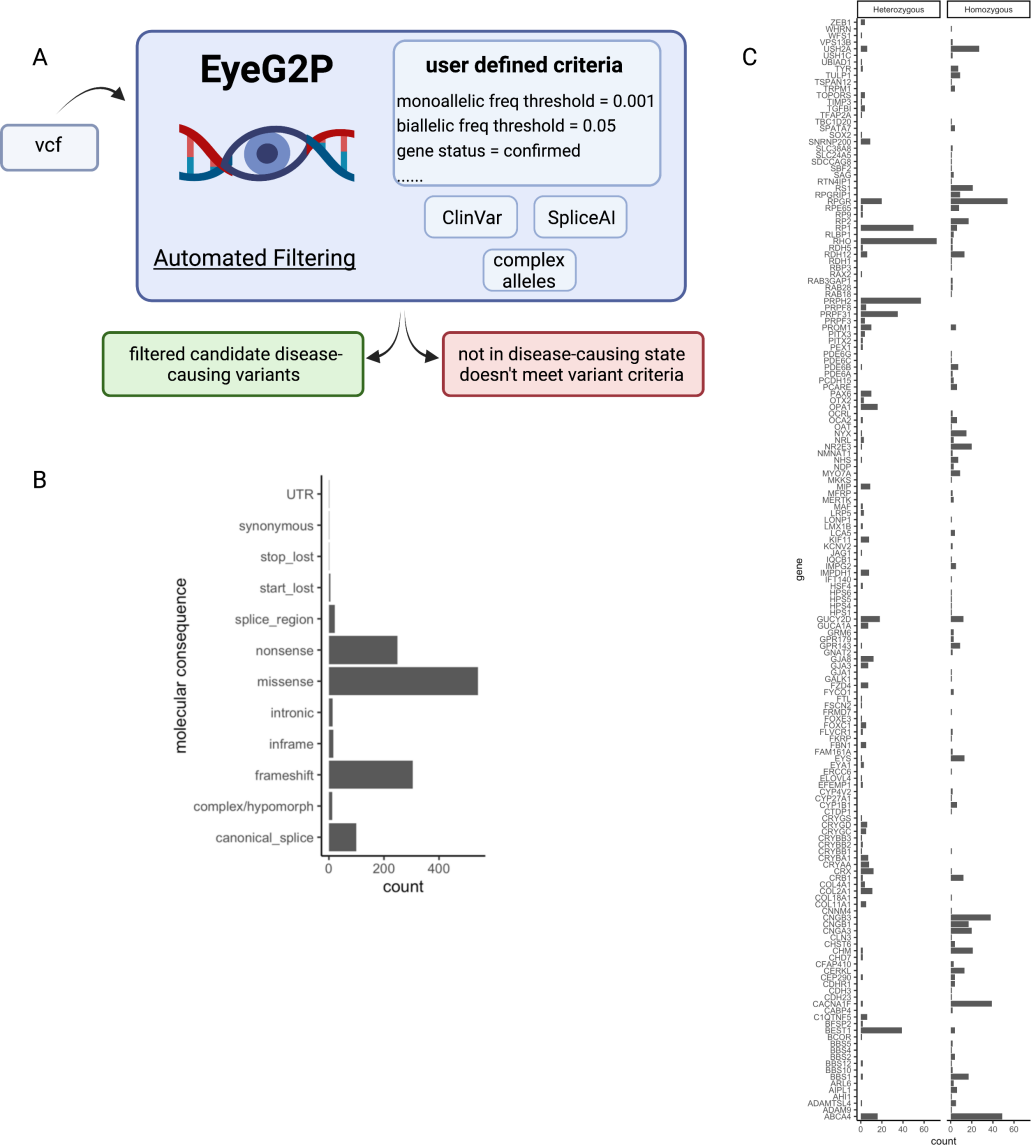
Molecular findings for 1228 individuals with a confirmed molecular diagnosis for inherited ophthalmic disorders. (A) Eye gene2phenotype (EyeG2P) is a plugin for the Ensembl variant effector enabling automated variant filtering and selection of variants in a disease-causing state (vcf, Variant Call Format). The specific requirements of variants to be retained can be set by the user and, through developments in the G2P software for the work described in this manuscript, can now include predefined lists of genomic variants, including pathogenic variants in ClinVar, variants predicted to impact splicing and complex alleles that comprised variants above the defined variant frequency threshold. (B) The predicted molecular consequences of 1267 variants identified as a cause of disease in 1228 individuals demonstrating the wide range of variant consequences that can be prioritised by EyeG2P (UTR, untranslated region). (C) The number of variants identified as a cause of disease in 166 genes by their proven zygosity. Hemizygous variants are included in the *Homozygous* display.

We assessed the capability of EyeG2P to identify molecular diagnoses in 1234 individuals who had previously undergone clinical diagnostic genetic testing at the UK North West Genomic Laboratory Hub. EyeG2P was able to prioritise the causal variants in 1228 of the 1234 study participants (99.5%, 95% CI=99.1% to 99.8%). The 1267 variants prioritised by EyeG2P were identified in 166 distinct genes and had diverse predicted molecular consequences (figure 1). These variants were detected in 497 individuals with autosomal recessive, 514 individuals with autosomal dominant and 217 individuals with X linked disorders. The six variants not identified by EyeG2P included 5’untranslated region (UTR) variants and intronic variants not prioritised by SpliceAI.

### EyeG2P increases precision over routine diagnostic testing

We performed a prospective comparison of the diagnostic capabilities of EyeG2P with routine diagnostic analysis in an additional cohort of 83 consecutively ascertained individuals with ophthalmic disease. A molecular diagnosis was confirmed in 33 of 83 cases (40%).

**Figure 2:**
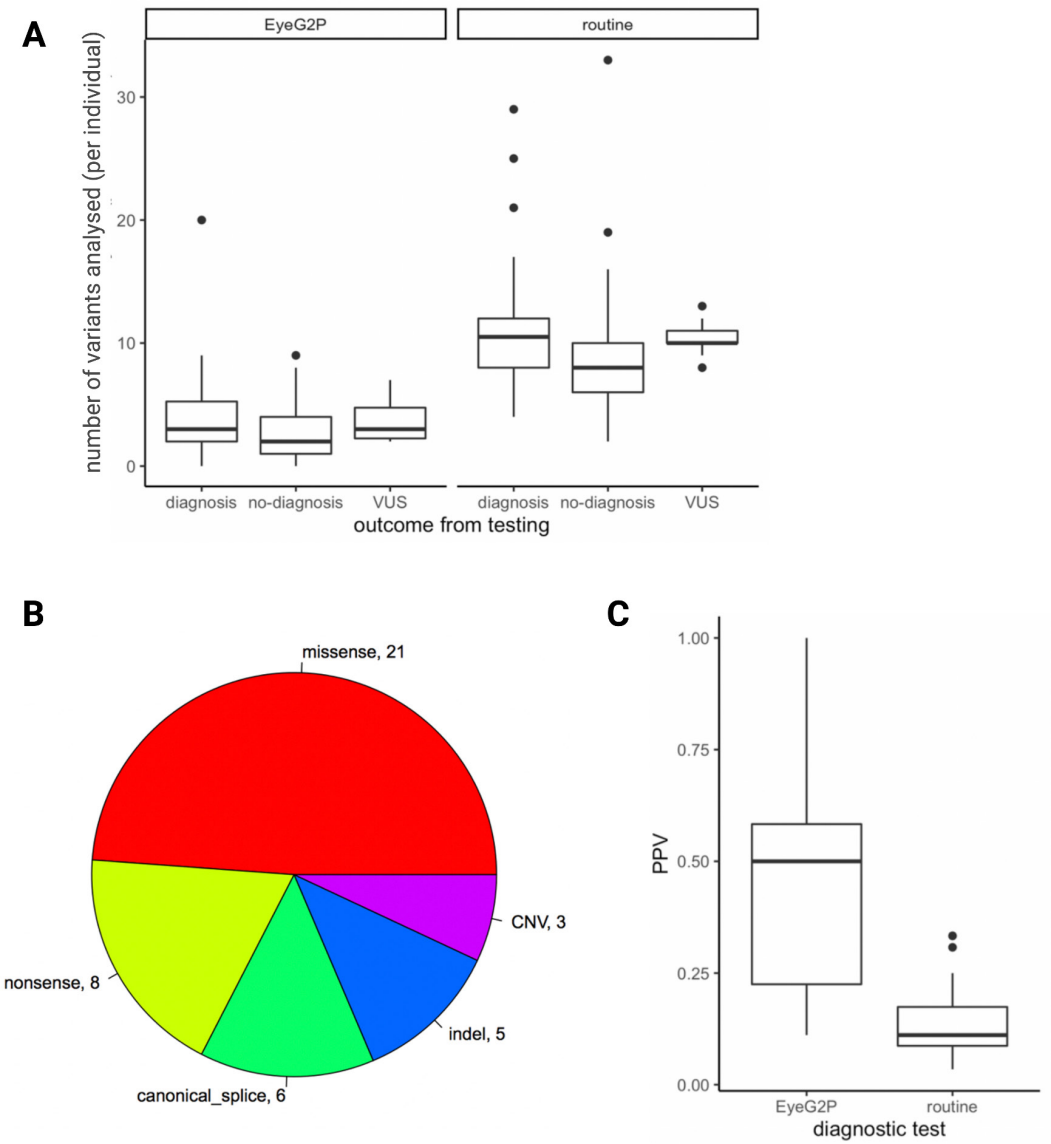
Direct comparison between EyeG2P and routine testing approaches identifies increased precision and efficiency of EyeG2P as a first-tier analysis approach. (A) The number of variants requiring analysis by clinical scientists for 83 individuals receiving genetic testing for inherited ophthalmic disorders (VUS, variant of uncertain significance). (B) Summary of the molecular consequences of pathogenic variants identified to underpin confirmed molecular diagnoses in 31 individuals. (C) The precision (PPV) of different testing approaches for 31 individuals receiving a diagnosis through both approaches. PPV, positive predictive value.

For 31 of 33 individuals (94%, 95% CI=84.0% to 99.5%), the confirmed molecular diagnosis was highlighted by EyeG2P. Importantly, EyeG2P allowed a reduction in the number of variants requiring analysis in each individual (mean=7.4; figure 2A). Disease-causing (pathogenic) variants were identified in 24 distinct genes (10 autosomal dominant, 3 X linked, 18 autosomal recessive disorders). The genomic variants underpinning these diagnoses included 3 CNVs (exonic deletions), 5 indels and 35 SNVs (figure 2B). Two cases had a confirmed genetic diagnosis of X linked retinitis pigmentosa as a result of variants in the orf15 region of the *RPGR* gene. These were not identified by EyeG2P as *RPGR* orf15 was excluded from EyeG2P analysis due to the limitations of short-read sequencing approaches in accurately identifying variants in this repetitive region—this is a limitation of the high-throughput short-read sequencing approaches applied, as variants in *RPGR* orf15 were not presented to EyeG2P for analysis.

In the remaining 50 cases in whom EyeG2P analysis did not identify a molecular diagnosis, we were also unable to identify a confirmed diagnosis through routine genetic testing approaches. Routine testing required an increased average analysis burden of 6.1 variants per individual (figure 2A). In 10 of 52 cases without a confirmed diagnosis, variants of uncertain significance were identified in a disease-causing state through the EyeG2P analysis.

We calculated the precision (positive predictive value) of genetic testing through EyeG2P and routine diagnostic genetic testing procedures for the 31 individuals who received a molecular diagnosis through both approaches. We observed a significant increase in precision in EyeG2P testing compared with default testing strategies (paired Wilcoxon rank-sum test, p<0.001), with an average increase in precision of 35% (figure 2C).

### EyeG2P effectively filters variants from whole genome sequencing

**Figure 3:**
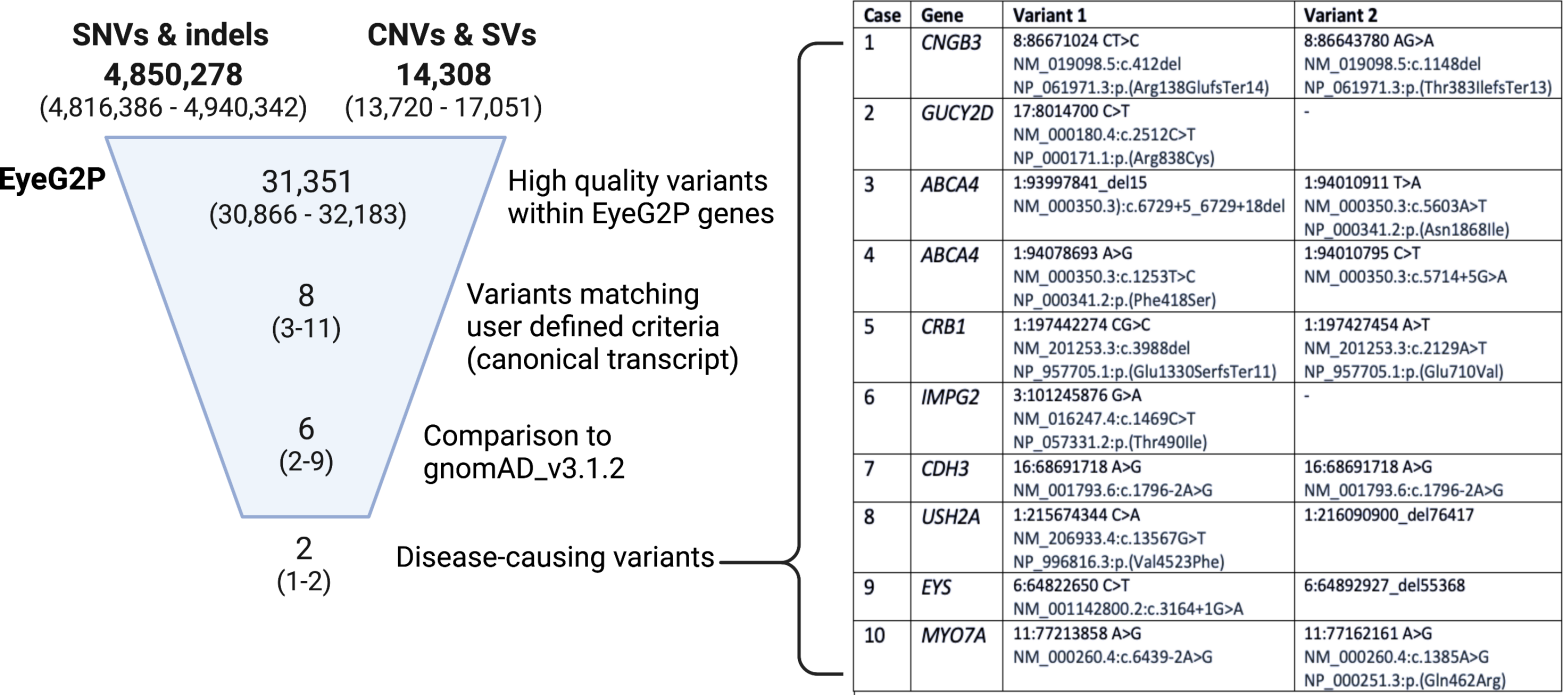
EyeG2P effectively prioritises pathogenic variation from whole genome sequencing data sets. The median and range of genomic variants identified across 10 individuals with whole genome sequencing analysis are shown at each stage of the filtering process. The complete list of clinically reported pathogenic and likely pathogenic variants prioritised by EyeG2P as a cause of inherited ophthalmic disorders is shown (genomic coordinates refer to the GRCh38 genome build). gnomAD, Genome Aggregation Database; indels, small insertion and deletion events; SNVs, single nucleotide variant; SVs, structural variants.

We assessed the capability of EyeG2P to prioritise genomic variants identified in 10 individuals with visual disorders recruited to the Genomics England 100,000 Genomes Project (figure 3). EyeG2P identified a genetic diagnosis for all 10 individuals. There was 100% concordance with variants that had been clinically reported through analysis (facilitated by UK NHS Genomic Medicine centres) for these individuals. The variants prioritised further demonstrated the diversity of variant types and variant impacts that can be prioritised by EyeG2P (figure 3), including CNVs, intronic variants and hypomorphic variants. The impact of EyeG2P on the analysis of genomic variation is substantial, reducing the number of variants for analysis to six, on average, while maintaining a 100% concordance in diagnostic yield.

Findings from EyeG2P were compared with the variants triaged and the overall diagnostic outcomes available through tiering and Exomiser variant analysis strategies applied during the 100,000 Genomes Project, which identified complete molecular diagnoses for 7 of 10 individuals. These strategies have developed over time and we also compared the findings with the variants triaged by an updated tiering process at Genomics England (table 1). Updates to tiering enabled a complete diagnosis for one additional individual through identification of a known pathogenic variant which was originally excluded from tiering due to allele frequency (figure 3, case 3), and highlighted CNVs in *USH2A* and *EYS* as TierA variants that were part of the molecular diagnosis for two of the individuals analysed (figure 3, cases 8 and 9). The average precision for known disease-causing variants was slightly increased through Genomics England tiering (median=28%, range=13%–50%; table 1) in comparison with EyeG2P (median=24%, range=11%–50%; table 1), although a remaining current advantage of EyeG2P is the capability to consider CNVs and SNVs simultaneously during variant prioritisation and thereby increased diagnostic yield in this cohort.

**Table 1:** Comparative performance of EyeG2P, Exomiser and Genomics England tiering approaches to identify disease-causing variants from whole genome sequencing data for 10 individuals with ophthalmic disorders

Case	Number of variants (molecular diagnosis)	Number of variants (EyeG2P)	Precision (EyeG2P, %)	Number of variants (updated tiering)	Precision (updated tiering, %)	Rank (Exomiser)	Precision (Exomiser, %)
1	2	8	25	5	40	1	7
2	1	8	13	6	17	1	1
3	2	11	18	7	29	Not found	–
4	2	4	50	7	29	2	1
5	2	7	29	4	50	1	0.3
6	1	9	11	6	17	2	0.2
7	1	3	33	8	13	2	2
8	2	7	29	3	33*	Not found	–
9	2	10	20	5	20*	Not found	–
10	2	9	22	4	50	2	0.2

Updated tiering numbers presented include tier 1, tier 2 and TierA variants.

*Complete molecular diagnosis not found.

## Discussion

Genomic testing for inherited ophthalmic disorders has been shown to have significant clinical utility.^4 10^ The expansion from single gene-based methodologies to the routine use of large gene panels, exome and whole genome sequencing approaches requires robust and precise variant filtering strategies that take into account phenotypic information. Moreover, the continual identification of novel disease-related genes requires analytical approaches that can evolve dynamically. Here, we describe EyeG2P, a filtering approach available as a plugin for the Ensembl Variant Effect Predictor.^16^ EyeG2P can be applied to any genomic variant data set in VCF format, including targeted gene panel, exome and genome data sets. We show that EyeG2P increases the precision and efficiency of genomic testing for inherited ophthalmic conditions over routine approaches for variant analysis.

The genetic basis of ophthalmic conditions is diverse and includes genes encoded by autosomes, sex chromosomes or mitochondrial DNA. This expands the initial assessments of G2P, which were largely focused on de novo variants causing dominant developmental disorders,^15^ to a group of disorders that display onset in childhood and in adults, and have notable levels of incomplete penetrance. Moreover, genomic diagnostic services available through high-throughput sequencing techniques are highly mature for ophthalmic disorders and therefore provide a useful and informative benchmark for comparison with G2P software. Following curation of over 1000 biomedical publications, we identified 667 relevant genes and determined the associated modes of inheritance, high-level molecular pathological mechanisms and phenotypic features. We have released these data as a publicly available resource that can be dynamically filtered and revised to best aid users’ requirements. For example, the recent elucidation of *DYNC2H1* as a cause of inherited ophthalmic conditions was not captured in our initial curation process, but this can be subsequently included in EyeG2P analysis through addition of a single data line to the released EyeG2P datafile.^28^ To ensure there is long-term support for ongoing gene curation approaches and consistency in the methodology undertaken, G2P/EyeG2P has joined the Gene Curation Coalition (https://search.thegencc.org/). This initiative will enable regular updates to the curated EyeG2P panel and enable coordinated collaborative efforts across gene curation initiatives.

Our ability to detect pathogenic genomic variants from high-throughput sequencing data sets has expanded in recent years to include a wide range of mechanisms, including large structural genomic variants,^29 30^ deletions and duplications within single genes (‘exonic deletions),^31 32^ variants deep within introns that may cause aberrant mRNA splicing,^33–36^ variants in regulatory regions^37–39^ and complex alleles that comprised combinations of genomic variants.^40 41^ This requires a high level of specialist knowledge. We found that, in addition to characterising novel exonic variants, EyeG2P is capable of prioritising these diverse types of pathogenic variation, achieving 99.5% sensitivity in comparison with routine analytical approaches (figure 1). As our knowledge of the specific genomic variants causing inherited ophthalmic conditions inevitably expands further, it will be possible for the user to adjust the analysis settings of EyeG2P to meet these requirements and/or to provide a list of specific variants for inclusion. Such approaches can be applied at scale, as demonstrated here for the analysis of whole genome data sets (figure 3). Of note, we also demonstrate the utility of updated versions of the Genomics England tiering approach to identify variants that would have been originally excluded during the 100,000 Genomes Project, as has been shown for incomplete and complete penetrance strategies applied to trios (https://www.genomicsengland.co.uk/blog/automated-variant-interpretation-2). This further demonstrates the value of continually evaluating and evolving variant analysis software and illustrates the potential benefits of reanalysis with updated bioinformatics approaches.

In conclusion, we demonstrate that EyeG2P can be effectively integrated with clinical diagnostic testing for inherited ophthalmic conditions to increase the efficiency of variant analysis. We show that EyeG2P reduces the variant analysis workload for clinical scientists and increases the precision of diagnostic testing. Moreover, EyeG2P can identify diverse genomic variants across the spectrum of genetically and clinically heterogeneous ophthalmic genetic conditions. We propose the application of EyeG2P as a first-tier analysis strategy for the diagnosis of inherited ophthalmic conditions from high-throughput genomic data sets.

## Data Availability

Shareable data is freely available through public resources, as specified in relevant sections of the manuscript, and is included in this manuscript.
